# NGS technology applied to melanoma cell lines

**DOI:** 10.1186/2051-1426-1-S1-P141

**Published:** 2013-11-07

**Authors:** Valeria De Giorgi, Zhi Xie, Jinguo Chen, Rongye Shi, Huizhi Zhou, Hui Liu, John Wunderlich, Michele Sommariva, Sara Tomei, Maria Libera Ascierto, Davide Bedognetti, Ena Wang, Francesco M Marincola

**Affiliations:** 1 Infectious Disease and Immunogenetics Section (IDIS), Department of Transfusion Medicine, Clinical Center, Trans-NIH Center for Human Immunology (CHI), National Institutes of Health, Bethesda, MD, USA; 2 Biometric Research Branch, Division of cancer treatment and diagnosis, National Institutes of Health, Rockville, MD, USA; 3 Surgery Branch, NCI, National Institutes of Health, Bethesda, MD, USA; 4 Weill Cornell Medical College in Qatar, Doha, Qatar; 5 Sidra Medical and Research Center, Doha, Qatar

## Background

High-throughput sequencing technology challenges the traditional histopathological classification of cancer, and proposes new taxonomies derived from global transcriptional patterns. They provided in-depth of new information that can reframe our understanding of human cancer biology. The novel observations that cancer phenotype characterized by immune effectors mechanisms are commonly observed during acute inflammation reaction. Several studies revealed associations between inflammatory status and a favorable natural history of the disease or a better responsiveness to cancer immune therapy. In the present study we applied RNA-seq analysis of 15 melanoma cells line, extensively classified (genetically, functionally and clinically) to explore the association between their constitutive pSTAT1 activation and the constitutive expression of genes associated with the regulation of the JAK/STAT pathway. With high resolution of sequencing information, we also attempt to identify the genetic determinants responsible for the phenotype (IRF-1, STAT1, Th1) associated with good prognosis and responsiveness to immunotherapy.

## Method

Rna-seq was performed using Solexa (Illumina GAII technology). CLC-bio workbench and deFuse algorithm were used for the alignment and for gene fusion analysis respectively.

## Results

Information about gene expression levels, splicing variants and fused gene were obtained. Novel fusion genes and mutations which could not be identified by conventional expression analysis were identified. Consistent tumor heterogeneity was observed across all 15 samples.

## Conclusion

Next generation sequencing technologies provides a more comprehensive view of cancer mutational landscapes and hereby a better understanding of their pathogenesis. This could be an open interesting perspective for new treatment approaches and clinical trial designs.

